# Epidemiological Survey on Water, Sanitation, and Hygiene (WaSH) in Uganda’s Karamoja Sub-Region, Using a KAP Questionnaire Within a One Health Framework

**DOI:** 10.3390/epidemiologia7020052

**Published:** 2026-04-07

**Authors:** Davide Ceccarelli, Silvana Diverio, Pier Giorgio Lappo, Carlo Ruspantini, Simon Peter Losike, Alma Rosa Pareschi, Maria Luisa Marenzoni

**Affiliations:** 1 Department of Veterinary Medicine, University of Perugia, Via San Costanzo 4, 06126 Perugia, Italy; davide.ceccarelli98@gmail.com (D.C.); silvana.diverio@unipg.it (S.D.); 2 Africa Mission-Cooperazione e Sviluppo, Via Cesare Martelli 6, 29122 Piacenza, Italy; coopdev.lappo@hotmail.it (P.G.L.); carlo.direzione@coopsviluppo.org (C.R.); 3 Cooperation and Development NGO (C&D), Lugugo Bypass, Plot 5, Kampala P.O. Box 7205, Uganda; coopdev.losike88@gmail.com (S.P.L.); alma.pareschi@gmail.com (A.R.P.)

**Keywords:** WaSH, KAP survey, Karamoja, livestock, infectious diseases, veterinary communication, water access, One Health

## Abstract

Background: The Karamoja sub-region of Uganda addresses significant challenges in water, sanitation, and hygiene (WaSH), deeply linked to public and environmental health and regional development. Objectives: This study applied a Knowledge, Attitudes, and Practices (KAP) survey within a One Health framework to assess WaSH conditions, hygiene behaviour, livestock management, and disease prevention in the Moroto and Napak districts. Methods: A total of 195 respondents were surveyed, providing insights into socio-demographic factors, hygiene practices, livestock management, and disease prevention. Results: Findings highlighted gender disparities, with women less likely to achieve good knowledge compared to men (OR = 0.04; *p* = 0.002), probably reflecting limited access to information in traditionally male-focused community settings, and their greater involvement in water collection tasks. Age significantly influenced WaSH knowledge, with older individuals (aged ≥ 30 years) showing higher odds of good knowledge (OR = 20.39; 95% CI: 2.74–151.83; *p* = 0.003), probably due to their roles in knowledge transmission within the community. Proximity to water sources shaped behaviours, with greater distances associated with improved attitudes (OR = 3.56; *p* = 0.002) but reduced hygienic practices (OR = 0.20; *p* = 0.01). Livestock ownership, particularly of small ruminants, strongly correlates with good hygiene knowledge (OR = 16.89; *p* = 0.02), probably due to interactions with veterinarians and authorities during vaccination campaigns. Integrated communication strategies, including community meetings, home visits, and radio outreach, were strongly associated with improved practices (e.g., home visits: OR = 30.78; *p* < 0.001). Conclusions: Despite progress, challenges such as water scarcity, waste management, and gender disparities persist. Improving water infrastructure, promoting equitable access, and integrating tailored communication strategies are essential for fostering sustainable development, health equity, and the empowerment of women in Karamoja.

## Simple Summary

In the Karamoja sub-region of Uganda, people face serious challenges related to water access, hygiene, and sanitation. This study explored water, sanitation, and hygiene (WaSH) conditions in the Karamoja sub-region, using a One Health approach, which considers the interconnection between human, animal, and environmental health. Through interviews with residents, information was collected not only on hygiene behaviours and access to safe water, but also on livestock ownership, animal management, and the role of veterinary communication. By integrating questions about both human and animal health, the study highlights how livestock and veterinary services can influence public health, especially in rural settings where animals play a central economic and cultural role. This approach provides a broader understanding of the challenges faced by communities and the complex dynamics affecting health and development in the region. The findings underline the importance of combining efforts across sectors and disciplines to improve health outcomes. Strengthening infrastructure, promoting hygiene education, and supporting animal health through accessible services are essential components of sustainable development in Karamoja.

## 1. Introduction

Karamoja is a sub-region in north-eastern Uganda with approximately 1.4 million inhabitants, bordering South Sudan to the north and Kenya to the east. It comprises nine districts: Abim, Amudat, Kaabong, Karenga, Kotido, Moroto, Nabilatuk, Nakapiripirit, and Napak. The region can be divided into an arid eastern zone characterised by predominantly nomadic pastoralism, a semi-arid central zone with an agro-pastoral system, and a western “green belt” with higher agricultural production.

Karamoja is the poorest region in Uganda, with about half of its population facing food insecurity [1,2]. Traditionally, its economy and social structure are based on pastoral activities, mainly cattle, followed by goats and sheep, and, in limited areas such as Abim district, camels. In this semi-arid environment, where agriculture is risky, livestock is essential for food security, particularly during droughts. Beyond subsistence, livestock also holds cultural and economic value, functioning as an investment for trade, acquisition of goods, and spiritual practices [3].

In recent decades, despite population growth, livestock ownership per capita has declined, leading to a redistribution of animals in which wealthier households accumulate most livestock at the expense of poorer families [4]. The decline is driven by multiple factors, including increased cattle raiding and security-related restrictions on herd mobility, which are traditionally guided by climatic conditions, and are now constrained by government disarmament programs that have confined livestock to protected kraals and restricted grazing to specific hours. These measures, often involving military control of herds, have contributed to substantial livestock losses due to diseases (particularly from animal overcrowding in confined spaces), restricted access to pasture and water, and added pressure on pastoral systems. Additionally, government and international initiatives to promote agriculture have not always fully accounted for the region’s climatic constraints, exacerbating these challenges faced by pastoral systems [4].

In this context, infectious diseases and the lack of veterinary services represent major constraints to livestock growth in Karamoja, causing estimated annual economic losses of approximately 92 million USD annually [5]. Major livestock diseases in Karamoja include trypanosomiasis [6], tick-borne diseases (TBDs), such as anaplasmosis, babesiosis, heartwater, and East Coast fever, transboundary animal diseases, including foot-and-mouth disease, contagious bovine pleuropneumonia, and peste des petits ruminants, as well as brucellosis and tuberculosis [7,8,9].

Water scarcity is a critical challenge in the semi-arid Karamoja region, particularly for livestock production. Available water sources include boreholes, windmills, and ponds for small ruminants and donkeys, and valley tanks, dams, rivers, and riverbeds for both small and large ruminants [4]. Although 79% of these water points were built in the last two decades, only 11% were specifically designed for livestock [10]. This limited water availability affects grazing practices, forcing livestock to remain closer to water sources during the dry season, leading to overgrazing and pasture degradation near these areas while more distant forage remains unused [10].

Traditionally nomadic pastoralists, the Karimojong have historically experienced relative protection from epidemic diseases such as Cholera and Measles for centuries [10,11,12]. However, especially since the early 21st century, increasing sedentary lifestyles [13,14], the creation of overcrowded settlements, poor immunity to epidemic diseases, and inadequate sanitary infrastructure have contributed to more frequent outbreaks of infectious diseases [15].

Water, Sanitation, and Hygiene (WaSH) is a public health framework aimed at ensuring access to safe drinking water, adequate sanitation, and appropriate hygiene practices, and represents a cornerstone for disease prevention, human dignity, and sustainable development [16,17]. Effective WaSH interventions are essential to prevent the spread of infectious diseases, especially in resource-limited settings such as the Karamoja Region, where water scarcity affects both human and livestock health. WaSH conditions and behaviours are commonly assessed through epidemiological household surveys and Knowledge, Attitudes and Practices (KAP) questionnaires, which are widely used to identify context-specific risk factors and barriers to effective prevention in low-resource settings [18,19]. Understanding the link between water management for humans and animals and changing occupational traditions is crucial to predict and prevent future emergencies and crises. From a One Health perspective, water quality and physical accessibility are vital to ensure the community’s well-being. Access to safe water varies considerably across Karamoja. While most families rely on boreholes, piped water, or springs, some still depend on surface water, as seen in Kaabong, where 24% of families used it as their primary source in 2017 [16]. Water scarcity emphasises the need for reliable water sources and proper water management practices. In Karamoja, women and girls are primarily responsible for water collection and face multiple challenges, including long distances to water sources, long queues, inadequate collection and storage equipment, water contamination, slippery roads during the rainy season, and risks of harassment, all compounded by seasonal variability and difficulties in accessing or pumping water [20]. When water is scarce, priority is often given to drinking and cooking, which may lead to the neglect of hygiene practices. Proper handwashing, for instance, significantly reduces the risk of diarrheal diseases by 23–40% [21]. Ensuring sufficient water supply, promoting hygiene education, and adopting appropriate water treatment and management practices are therefore essential to prevent waterborne diseases [21].

This study investigates the complex relationship between human and animal health in relation to WaSH practices in the Moroto and Napak districts of the Karamoja sub-region, where WaSH conditions may differ from other districts due to variations in socio-economic factors, water availability, and infrastructure. Using data from a KAP questionnaire, the study aims to identify key challenges and propose targeted interventions to improve public health and well-being from a One Health perspective, addressing the interconnected needs of humans, livestock, and the environment.

## 2. Materials and Methods

### 2.1. Study Design and Area

The study was conducted in the Moroto and Napak districts from January to March 2023. Karamoja is a semi-arid area characterised by recurrent droughts, high climate variability, and chronic water scarcity, conditions that strongly influence livelihoods, health outcomes, and access to basic services. This region is predominantly inhabited by pastoral and agro-pastoral communities, with close interactions between humans, livestock, and the environment, making it particularly relevant for WaSH investigations within a One Health framework. Moroto and Napak were selected as focus areas because they belong to the same socio-cultural and ecological context of the Karamoja sub-region. While Moroto district hosts the main urban centre of the region, it also includes extensive rural areas and villages characterised by pastoral and agro-pastoral livelihoods; Napak district is predominantly rural, with dispersed settlements and limited infrastructure, reflecting the typical settlement pattern of the semi-arid region. This combination allowed the assessment of WaSH conditions and behaviours across both urban–peri-urban and rural pastoral contexts within the same ecological and cultural setting. These districts were also selected within the broader framework of the ALL IN ONE project, funded by the Italian Agency for Development Cooperation (AICS), a multiple-partner project, led by the Africa Mission–Cooperation and Development (Africa Mission-C&D).

This study was carried out through a collaborative effort involving the Department of Veterinary Medicine of the Perugia University (Italy) for the scientific planning and support, and the Africa Mission–C&D, a non-governmental organization with over 50 years of experience in Karamoja, that provided logistical support, including interpreters and transportation, and played a key role in adapting the KAP questionnaire (see below) to the local context, leveraging their strong community trust and operational expertise. Within the broader framework of the ALL IN ONE project, this survey represented a key initial activity, designed to collect baseline data on WaSH conditions in Moroto and Napak, intended to provide a solid evidence base for interpreting the local context and to inform targeted interventions, ensuring that subsequent project actions would be tailored to the specific needs and challenges of these districts.

### 2.2. KAP Questionnaire

The KAP questionnaire was designed to gather essential epidemiological information on health and hygiene behaviours (WaSH topics) among the Moroto and Napak population. KAP surveys represent a widely used and standardised tool in WaSH research, particularly in low-resource settings, for assessing context-specific risk factors and behavioural determinants related to disease prevention [18,19]. It was structured into four main areas to explore different aspects of daily life and practices: (1) demographic information, with details about age, gender, ethnic affiliation, residence, education level, occupation, and household size, family role; (2) Knowledge, with the evaluation of respondent knowledge on waterborne diseases, their transmission routes, hygiene practices, and the sources of health-related information they relied on; (3) Attitude, investigating attitudes towards clean water, sanitation, and hygiene, including perceptions about open defecation, the benefits of latrine use, and the willingness to change existing habits; (4) Practices, exploring practices related to water use, purification methods, sanitation facilities, handwashing, and the disposal of faeces of children. Special attention was given to livestock and water management for animals. Additional questions explored water availability for livestock, animal management practices, and preventive measures against zoonotic diseases, recognising the integral role of livestock in the local economy and culture.

The questionnaire comprised 52 core questions and 26 optional questions, based on the initial answers of the respondents. For analysis purposes, the questions were also grouped into six thematic indicators: (a) water supply and management: various aspects of water sources (type and reliability of water sources, collection, storage, and treatment methods used) were analysed to understand how it impacts daily life; (b) hygiene: to understand personal hygiene and household hygiene practices used; (c) sanitation: to understand type and use of sanitation facilities; (d) waste management: to observe locations and how waste was disposed of; (e) communication and knowledge of infectious diseases: to identify the preferred communication methods, assess which have the greatest impact on knowledge, and evaluate general awareness of infectious diseases and their prevention; (f) livestock: to analyse the possession of animals and their management, regarding both water availability and the prevention of diseases. The complete questionnaire is available as Supplementary Material (Questionnaire S1).

The design and contextual adaptation of the questionnaire were carried out in collaboration with Africa Mission-C&D, whose longstanding experience in the region provided invaluable insights. The questions were made to ensure they were culturally sensitive, easily understandable, and appropriately framed for the local context. Practical considerations such as the average duration of interviews, potential privacy concerns, and the cultural interpretation of certain terms were also carefully addressed.

To enhance validity and reliability, the questionnaire underwent a pilot testing phase with a small group of 10 participants prior to data collection. This process allowed the identification and correction of structural or linguistic issues and ensured internal coherence and cultural appropriateness of the instrument. Data were collected through face-to-face interviews conducted by trained local staff in the local language, reducing the risk of misinterpretation and non-response bias. Standardized data collection procedures and the use of logical branching within the digital questionnaire further enhanced the consistency and reliability of the collected data.

### 2.3. Ethical Considerations

The study adhered to ethical guidelines for research involving human participants, ensuring the privacy and rights of all respondents. The questionnaire was conducted anonymously, and no personal or sensitive data were collected. Participation was voluntary, and respondents were informed about the purpose of the study, how the data would be used, and their right to decline any question or withdraw at any time. Local interpreters were trained to explain this information clearly to participants before starting each interview.

To ensure cultural sensitivity, questions that could be perceived as intrusive or inappropriate were reviewed and modified with input from colleagues familiar with the Karimojong culture. Participants were also informed that the study aimed to collect data on WaSH-related challenges and that its scope did not include direct resolution of those issues. These measures were designed to maintain trust and respect while upholding the anonymity and dignity of all participants.

Given the non-invasive nature of the study and the absence of personally identifiable information, formal ethical approval was not initially required, and the research was conducted in line with local and international ethical standards. Retrospective approval for data analysis and publication was subsequently obtained from the Bioethics Committee of the University of Perugia (protocol n. 128398, ethics approval document n. 22/2025), in line with institutional and international ethical standards.

### 2.4. Sampling Method

The aim was to interview residents from various villages scattered across the Moroto and Napak districts, as well as residents from the city of Moroto, the capital of Karamoja, to obtain a comprehensive picture of the situation. A Simple Random Sampling method was used to obtain a representative sample, with data points relative to the number of households in each village to ensure that the data were statistically significant and reflective of the wider community. However, the selection process was affected by the ongoing armed conflicts, which restricted access to certain areas, and by the willingness of the residents to participate. In cases where participants declined to answer, nearby villages were selected as alternatives to maintain the sample size and geographical diversity.

### 2.5. Questionnaire Administration

The questionnaire was administered through face-to-face interviews conducted by trained local staff from Africa Mission-C&D. Interviews were conducted in the local language to ensure comprehension and accurate responses. The data collected were recorded and later translated into English for analysis. The translation from the local language to English did not require formal back-translation, as the aim was to ensure comprehension of each question rather than to validate complex conceptual constructs. The questionnaire had been culturally adapted beforehand, and interviewers provided direct word-by-word translation to preserve meaning.

### 2.6. Data Collection

All the information was collected using the structured questionnaire, gathering both qualitative and quantitative data. The questionnaire was then transferred to the data collection software Kobo Collect (version 2021.2.4, KoboToolbox, Harvard Humanitarian Initiative, Cambridge, MA, USA) to facilitate its administration on smartphones and tablets. The questionnaire was programmed with logical branching within the Kobo Collect software, ensuring that relevant follow-up questions appeared based on the previous answers of the respondents (e.g., if a participant indicated not owning livestock, subsequent questions related to livestock management would be skipped). This logic aimed to make the interview process more seamless and streamlined.

### 2.7. Data Analysis

Descriptive statistics were used to summarize the data, providing an overview of the demographic, knowledge, attitudes, and practices (KAP) variables. To explore associations, the responses related to knowledge, attitudes, and practices were scored based on their appropriateness for prevention and correctness. Responses were coded as 1 (correct) or 0 (incorrect), creating binary variables to reflect the suitability of knowledge, attitudes, and practices of the respondents concerning WaSH and livestock management. The variables “correct” were further categorised into two levels: sufficient or good, to differentiate the degree of appropriateness. Each independent variable was analysed individually to assess its association with the outcome, which was the appropriateness of knowledge, attitudes, and practices in two levels (sufficient and good), to ascertain their relationship using the χ^2^ test.

The variables scoring *p* ≤ 0.2 in the univariate model were included in a multivariable model, where the outcome of interest was the appropriateness of knowledge, attitudes, and practices in two levels: sufficient and good. This approach allowed for the identification of key factors influencing the appropriateness of WaSH-related behaviours. Predictors included demographic variables such as age, gender, education level, and occupation, along with additional variables: type of animals owned, distance from the water source, preferred communication methods, ownership of electronic devices, access to the Internet, ability to speak English, and awareness of hygienic practices. The analysis aimed to highlight areas for targeted interventions by exploring how different predictors influenced the levels of knowledge, attitudes, and practices. By differentiating responses into sufficient and good levels, the study provided deeper insights into the variation in WaSH-related behaviours within the population. Logistic regression was employed to evaluate these associations, with adjusted odds ratios (ORs) and 95% confidence intervals (CIs) calculated to quantify the strength and direction of relationships. Statistical significance was set at *p* < 0.05. All analyses were performed using the open-source R statistical software (version 4.2.2, R Foundation for Statistical Computing, Vienna, Austria).

## 3. Results

The results provide a comprehensive overview of the different sections of the questionnaire, with descriptive statistics that summarise the main patterns, while multivariate statistical analyses explore associations between knowledge, attitudes, and practices and key demographic and contextual variables in the Karamoja sub-region.

### 3.1. Descriptive Statistics

#### 3.1.1. Demographic Information

The demographic questions collected detailed information on each individual’s role, gender, age, place of residence, ethnic affiliation, education level, job, and the number of children they had. The survey involved a total of 195 individuals, including 99 women and 96 men, aged between 16 and 80 years. These participants were from various locations, with 158 from the villages of Moroto and Napak districts and 37 from Moroto City.

Regarding education, a slightly higher percentage of women (25%) reported never having attended school compared to men (21%). The majority of both women and men completed primary or secondary school, with only a small percentage attending university. There was a stark contrast between those living in Moroto city and those in villages, with the city dwellers having higher education levels. Table 1 synthesises these data.

Employment patterns also varied by gender, with 22.2% of women reporting unemployment compared to 12.5% of men. The types of employment differed, with women mostly engaged in small-scale businesses, selling local brew, tailoring, and agriculture, while men were involved in small-scale businesses, agriculture, and local administration. Table 1 synthesises these data. This data is the basis for understanding variations in WaSH practices and their determinants across different population subgroups.

#### 3.1.2. Water Supply and Management

Most of the population (over 95%) uses safe water sources, primarily hand pumps/boreholes (71.8%) and public taps (20%). However, 4.6% still rely on unsafe sources like rivers.

When it comes to the reliability and accessibility of water sources, about 46% of respondents indicated that their primary water source is not accessible or reliable throughout the year, citing issues like borehole breakdowns (49%) and water depletion during the dry season (37%). Other challenges include restricted access due to private control, rusted equipment, and conflicts over water collection.

Consequently, about 34.4% have a secondary water source, mainly a second borehole, while 49.2% have never used an alternative source. Table 2 resumes these answers in detail.

On average, 74% of respondents travel less than 1 km to collect water, while 25% must travel over 1 km. Water transportation is primarily carried out by women (63.3%), highlighting the significant role they play in managing household water needs. Table 2 shows these data. Despite the considerable distance travelled by some to access water, most families make the journey about 2.8 times per day on average, regardless of the distance.

Approximately 97% of residents use jerry cans to transport water, and 74% store it in the same container at home. Water storage practices vary, with 9% transferring the water to jars, 8% to buckets, and 9% to larger containers.

Water treatment practices are also a concern. Around 60% of respondents do not treat their water before consumption, mainly because they perceive it as unnecessary or safe as is. Of those who do treat their water, 33% boil it, 5% use specific treatment products, and 3% let it stand and settle. The treatment practices vary depending on the source, with a higher percentage of those using boreholes and public taps choosing to boil the water, while those relying on surface water are less likely to treat it, often due to a lack of access to treatment products.

Of all those who changed their water source in the past 10 years, 92% noted that they have had more free time since then. The positive response rate was 100% among women and 83% among interviewed men. Among these individuals, 26% started a new activity, primarily kitchen gardening, small local trade, or increased cleanliness of their homes or clothing. When asked if they believed that the social condition of the family had improved since changing the water source, 66% responded positively.

#### 3.1.3. Hygiene

Hygiene practices among respondents show a generally positive trend in water container maintenance and handwashing habits. A significant majority (97.9%) of respondents clean their water containers, with 56.9% doing so at least once a week. The most common cleaning method involves using sand and stones, often combined with soap. Details of these answers are reported in Table 3.

**Figure 1:**
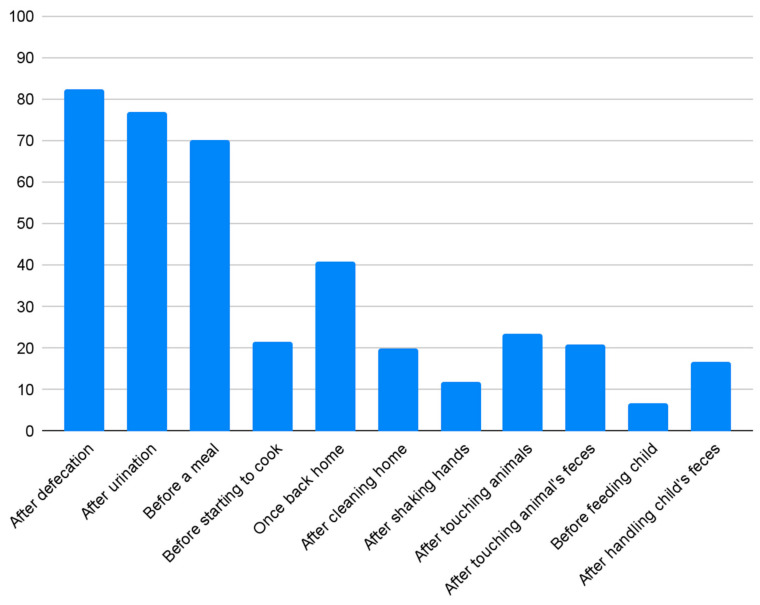
Percentages of people washing their hands and activities requiring it.

Regarding handwashing, 55.9% of the respondents claim to have a container in their homes exclusively used for this purpose, with 18.5% owning a specific pouring device. The remaining 44.1% use the same container for other functions. Of the overall population, 87.2% report using soap for handwashing. Among this group, 37.4% use ashes when they run out of soap. Finally, 6.7% use ashes exclusively, and the remaining 6.2% use only water. Those who do not use soap were asked for a reason, and in 92% of cases, the response was due to economic reasons. The remaining 8% prefer to use ashes, considering it a traditional method of handwashing. Respondents were asked to reflect on their daily routines and provide open-ended answers regarding the times of day when they wash their hands (Figure 1). A total of 82.6% state that they wash their hands after defecation, and 76.9% after urination. In a culture where eating food with hands is common, only 70.3% wash their hands before a meal, and 21.5% before starting to cook. Around 41% claim to wash their hands once they return home, 20% after cleaning the house, and 11.8% after shaking hands with someone. Among those who own animals, 23.4% state that they wash their hands after touching them, and 20.8% after touching their excrement. Among those with children under the age of 5, 6.7% wash their hands before feeding the child, and 16.7% after handling their faeces.

#### 3.1.4. Sanitation

Sanitation practices vary between urban and rural areas, with 71.8% of respondents using both private and public latrines. In cities, public latrine usage is higher (59.5%), whereas in villages, private latrine usage is more common (40.5%). Open defecation is still practised by 28.2% of the population, with a higher prevalence in villages (33.5%) compared to cities (5.4%), as detailed in Table 4.

#### 3.1.5. Waste Management

Waste management practices are largely informal, with 52.3% of the population burning waste near their homes. A smaller portion of waste is disposed of in common (14.9%) or private pits (9.7%), with some using designated (4.6%) or undesignated (14.4%) open areas. A minority (3.1%) dumps waste into rivers, highlighting the need for improved waste management infrastructure. Table 5 summarizes this information.

#### 3.1.6. Communication and Knowledge of Infectious Diseases

Literacy rates are significantly higher among younger generations, with 88% of male children and 67% of female children able to read, while older generations, particularly grandmothers, show much lower literacy rates (14%). Details of the percentages of individuals with at least primary education are reported in Table 6.

Ownership of electronic devices is relatively common, with 84% of households owning a mobile phone and 55% owning a radio. However, only 32% of mobile phones have internet access, limiting the effectiveness of digital campaigns. Despite this, radios remain the most effective communication tool, preferred by 56% of respondents for receiving health and hygiene information. Community meetings (45%) and home visits (33%) also play crucial roles in disseminating information.

Considering the importance of community meetings in Karimojong culture, the respondents were asked if they had recently participated in a community meeting on the topic of WaSH, and an overwhelming 85% responded affirmatively. The most discussed topics in these meetings included the importance of personal hygiene (73.5%), maintaining proper cleanliness of their homes (65.7%), and the importance of using latrines (60.8%). Other topics included diseases caused by poor WaSH practices (16.3%), the importance of handwashing with soap (15.1%), proper waste disposal (11.4%), and water treatment before consumption (10.8%). A small percentage (3.6%) also mentioned the importance of cutting nails, especially to avoid contaminating food.

While most respondents have some knowledge of disease transmission and prevention, significant gaps remain. For example, 24.6% of respondents do not recognize contaminated water as a cause of diarrhoea, and 29% do not believe that a deceased person can transmit diseases. Additionally, misconceptions exist regarding the role of animals in disease transmission; 15% believe dogs cannot transmit diseases, 22% ruminants cannot transmit diseases and 30% believe chickens are also safe.

The respondents were then asked about their knowledge of diarrhoea, a common issue in the region, particularly regarding its causes. The vast majority (84.6%) knew that diarrhoea can result from consuming contaminated or undercooked food. Specifically, 32.3% mentioned contamination by flies, especially in relation to open defecation (8.7%) or eating in unsanitary environments (7.7%). Other reported causes included not washing hands at critical times (16.9%) and not trimming nails regularly (8.2%). Only a quarter (24.6%) of respondents believed that diarrhoea could also be caused by drinking contaminated water. Additionally, 8.2% mentioned the possibility of contracting diarrhoea through contact with an infected person, while 1.5% referred to contact with a sick animal.

In an open-ended question, the respondents were asked to name the diseases they feared the most. The top responses included COVID-19 (53.8%), cholera (52.8%), HIV (41%), Ebola (29.7%), tuberculosis (25.6%), and malaria (19%). Other mentioned diseases included other sexually transmitted diseases (8.2%), trachoma (5.6%), influenza (5.1%), typhoid fever (3.6%), and brucellosis (3.1%). A significant 87% of respondents reported having received information from the government, local authorities, international organisations, or through community meetings regarding the prevention of the infectious diseases they feared most. The most common communicated preventive measures included handwashing at key times (52.3%), maintaining distance from individuals who appeared ill or suspicious (45.6%), covering the nose and mouth (37.4%), maintaining good personal hygiene (21.5%), proper cooking of food (18.5%), appropriate food storage (10.3%), and treating water before consumption (10.8%). However, 39% of respondents were unable to mention any preventive measures. The preventive measures considered useful by the interviewees and related to the feared diseases are reported in Table 7.

#### 3.1.7. Livestock

Among the 195 respondents surveyed, only 39% owned animals. Those who reported not owning any livestock often cited recent raids or lack of funds to purchase them as reasons.

Chickens were the most commonly owned livestock (61%), followed by goats (56%) and cattle (45%). Ownership of other animals, such as sheep, ducks, turkeys, and rabbits, was significantly lower (Table 8). Livestock ownership was higher in rural areas than in urban settings, and it was often concentrated among a small percentage of wealthier families.

The primary water sources for livestock included surface water, protected springs, boreholes, and public taps. Cattle owners predominantly used surface water (40%), while small ruminants and poultry owners relied more on boreholes. Full percentages for each livestock category and water source type are reported in Table 8. The distance to water sources varied: boreholes and public taps were generally closer to households, whereas protected springs and surface water sources were often farther away, sometimes more than 10 km (see Table 8).

The availability of reliable water sources was not a strong incentive for non-owners to acquire livestock, as only 24% indicated they would consider purchasing animals if a new water source became available. The main barriers to livestock ownership included high costs, lack of interest, urban living conditions, and the risks associated with raids.

Among those who owned livestock, 77% reported receiving information from local authorities on how to prevent the spread of diseases. Vaccination (56%), isolation of sick animals (40%), and cleaning their resting places (30%) were the most commonly known and applied preventive measures. However, the awareness and implementation of these measures were notably higher among those who had received official communication compared to those who had not.

**Figure 2:**
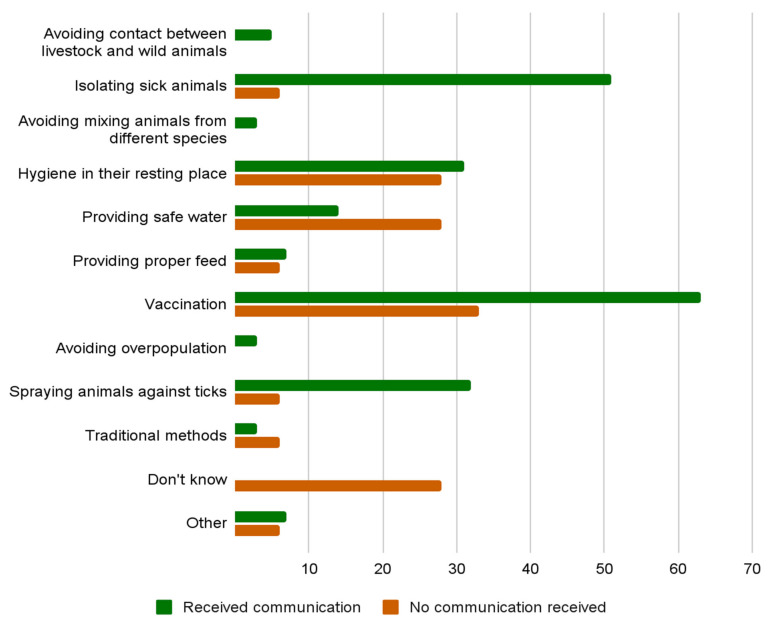
Awareness of preventive measures against infectious diseases in livestock: a comparison between owners who received communications from authorities and those who did not.

Figure 2 reports the percentages of communicated or non-communicated preventive measures against infectious diseases in livestock.

### 3.2. Multivariate Analysis

Different multivariable models identified significant factors influencing sufficient and/or good levels of knowledge, attitudes, and practices related to WaSH among the surveyed population.

#### 3.2.1. Sufficient and Good Level of Knowledge

The likelihood of achieving sufficient knowledge was positively associated with communication methods and employment status. Participants who reported receiving home visits as a primary communication method were nearly twice as likely to exhibit sufficient knowledge compared to those who relied on other communication methods (OR = 1.96, 95% CI: 1.06–3.62, *p* = 0.031). Similarly, having stable employment more than doubled the probability of sufficient knowledge (OR = 2.22, 95% CI: 1.15–4.30, *p* = 0.017). Table 9 provides an overview of the predictors for sufficient knowledge.

Good knowledge levels were strongly influenced by access to technology and livestock ownership. Participants with Internet access had 25 times higher odds of achieving good knowledge (OR = 25.04, 95% CI: 3.17–197.68, *p* = 0.002), and those using the Internet at home saw an even greater likelihood (OR = 69.80, 95% CI: 4.28–1138.04, *p* = 0.003).

Sheep ownership was also positively associated with good knowledge, with owners showing significantly higher odds (OR = 16.89, 95% CI: 1.54–185.64, *p* = 0.021). Conversely, being a woman was associated with a reduced likelihood of good knowledge (OR = 0.04, 95% CI: 0.006–0.30, *p* = 0.002), and higher education also had a negative association (OR = 0.01, 95% CI: 0.001–0.14, *p* < 0.001) (see Table 9).

#### 3.2.2. Sufficient and Good Level of Attitudes

Sufficient attitudes were influenced by access to water and awareness of hygienic practices. Households located farther from their primary water source (≥1 km) were significantly more likely to exhibit sufficient attitudes (OR = 3.56, 95% CI: 1.57–8.11, *p* = 0.002). However, greater awareness of hygiene practices showed an unexpected inverse association (OR = 0.32, 95% CI: 0.13–0.83, *p* = 0.020) (Table 10).

Good attitudes were more likely among individuals with higher education (OR = 6.89, 95% CI: 3.12–15.20, *p* < 0.001) and those owning livestock (OR = 6.96, 95% CI: 2.90–16.70, *p* < 0.001). Participation in community meetings also significantly improved attitudes (OR = 2.34, 95% CI: 1.12–4.90, *p* = 0.024).

In contrast, urban residence in Moroto City (OR = 0.10, 95% CI: 0.03–0.35, *p* < 0.001) and cattle ownership (OR = 0.14, 95% CI: 0.05–0.43, *p* < 0.001) were associated with lower likelihoods of good attitudes (Table 10).

#### 3.2.3. Sufficient and Good Level of Practices

Sufficient practices were influenced by gender, communication tools, and water accessibility. Men were more likely than women to achieve sufficient practices (OR = 0.36 for women, 95% CI: 0.16–0.83, *p* = 0.017). Radio ownership significantly increased the likelihood of sufficient practices (OR = 3.53, 95% CI: 1.45–8.59, *p* = 0.005). Additionally, a greater distance to water sources (≥1 km) was strongly associated with sufficient practices (OR = 6.10, 95% CI: 2.72–13.68, *p* < 0.001) (Table 11).

Good practices were positively associated with several factors, including home visits, which emerged as the strongest predictor (OR = 30.78, 95% CI: 8.07–117.42, *p* < 0.001). Owning a radio (OR = 15.72, 95% CI: 4.04–61.20, *p* < 0.001), Internet access (OR = 21.30, 95% CI: 6.07–74.70, *p* < 0.001), and poultry ownership (OR = 4.61, 95% CI: 1.49–14.22, *p* = 0.008) were also significant positive predictors.

Gender differences were noted, with women showing higher odds of good practices compared to men (OR = 4.49, 95% CI: 1.43–14.08, *p* = 0.010). Higher education further increased the likelihood of good practices (OR = 4.17, 95% CI: 1.36–12.75, *p* = 0.012). In any case, a greater distance to water sources was negatively associated with good practices for all respondents (OR = 0.20, 95% CI: 0.06–0.70, *p* = 0.011) (Table 11).

## 4. Discussion

This study investigated the current situation in the WaSH sector of the Karamoja sub-region of Uganda, examining quality of life, hygiene habits, and communication methods, and adopting a One Health approach to identify both achievements and persistent challenges.

### 4.1. Water Accessibility and Management

The use of safe water sources is consistent with previous findings [16], with only 4.6% of the population relying on unsafe sources. However, about 25% still walk more than 1 km to access water, highlighting the ongoing challenges of water accessibility. This aligns with findings showing that greater distances from water sources are positively associated with sufficient attitudes toward hygiene but hinder good hygiene practices. The role of women in water collection, constituting 63.3% of water gatherers, underscores the burden placed on them and the necessity of empowering women through infrastructure improvements. Previous studies have documented that women and girls often bear the physical and time burden of water fetching, which can limit their participation in education and economic activities [22,23]. Efforts to rehabilitate wells and build new ones closer to households could alleviate this burden while also promoting socio-economic opportunities. All the respondent women (100%) who accessed nearer water sources reported increased free time, which they used for activities like small-scale trade, gardening, and household improvements, leading to greater societal roles and contributing to achieving the gender equity goal promoted by the WHO [24]. This finding supports previous evidence linking improved water accessibility to women’s empowerment and socio-economic participation [22,23].

### 4.2. Hygiene Practices

Hygiene practices show both progress and gaps. While 87.1% of respondents reported using soap for handwashing, significant gaps remain in practices for preventing infections, such as washing hands before cooking or eating. This is particularly concerning given the high risk of gastroenteric diseases associated with contaminated food and water. Only 23.4% of the respondents owning livestock wash their hands after handling animals, posing risks for zoonotic disease transmission (e.g., bacterial and viral zoonosis, zoonotic parasites). Similar low compliance has been documented in rural communities of Ethiopia, where handwashing after handling live animals was reported by less than one-third of respondents, and was strongly associated with education level and awareness of zoonotic risks [25].

Gender was shown to play a significant role in hygiene practices, with women being less likely to practice sufficient hygiene but more likely to excel in good hygiene practices once barriers are overcome. These barriers often include limited and irregular access to clean water and soap, exacerbated by the extensive time women spend fetching water, which directly reduces the frequency of hygienic behaviour [23,26,27]. In addition to infrastructural constraints, socio-cultural factors, such as gender roles, traditional norms, and low risk perception, can hinder the adoption of recommended practices [26,27]. Tailored interventions that address both physical and cultural barriers are therefore essential, particularly among women and those with lower literacy levels.

Urban residence was negatively associated with good attitudes towards hygiene, possibly reflecting greater reliance on available services, in contrast to rural settings, where self-sufficiency and preparedness are often essential for maintaining good hygiene. This dynamic is consistent with findings from a recent study in Tanzania, where rural students demonstrated slightly higher WaSH attitude scores compared to their urban peers [28].

The role of communication tools, such as home visits, radio broadcasts, and internet access, has also been identified as crucial for promoting awareness and triggering behaviour change. While mobile phones have emerged as the most effective channel for influencing health-related behaviours in sub-Saharan Africa, radio and television remain particularly important among people, and especially women, with lower education levels, where access to digital technologies is still limited [29].

### 4.3. Sanitation and Waste Management

Our survey showed that open defecation was reported by 28.2% of respondents, indicating a marked improvement compared to the 73% recorded in the Karamoja sub-region in 2017 [16]. The increased use of public and private latrines signifies progress, yet substantial challenges remain in waste management practices.

Burning waste, reported by 52.3% of respondents, is also widespread in other rural African settings and is associated with adverse health outcomes, including respiratory diseases, soil and water contamination, and environmental pollution [30]. It may contribute to pollution and antibiotic resistance, particularly through the improper disposal of veterinary treatments [31]. Addressing these interconnected risks requires a One Health approach. In this context, the prevalence of informal waste management practices underscores the need for further and complementary research to investigate potential environmental and water-quality risks, including the chemical safety of drinking-water sources, to a more comprehensive understanding of WaSH-related health threats within a One Health framework.

### 4.4. Communication and Knowledge of Infectious Diseases

Effective communication plays a pivotal role in improving knowledge and practices. Home visits are the most effective for improving sufficient knowledge, while Internet access and community meetings significantly enhance good hygiene and sanitation practices, showing that efficacy is reached only when more communicational approaches have been undertaken. Evidence from our survey confirms that multi-channel communication strategies are associated with better outcomes, in line with previous studies demonstrating that diversified communication approaches enhance message reach and retention in rural African settings [29,32].

Gender-related differences in knowledge and practices were evident, with women showing lower odds of achieving sufficient knowledge compared to men. This may be partly explained by traditional gender roles, which can limit women’s participation during community meetings, where women, although present, may have fewer opportunities to speak or to be targeted by communication efforts, potentially limiting their direct access to critical information [26,27]. Addressing this gap requires targeted interventions that account for women’s communication preferences and access constraints.

Additional findings indicate that having a job doubles the likelihood of sufficient knowledge, suggesting a possible dual interpretation: increased knowledge may enhance job opportunities, or, otherwise, employment may facilitate access to knowledge.

Higher levels of formal education correlated positively with attitudes and practices, as expected and reported in existing literature [33], but were, surprisingly, inversely associated with good knowledge regarding WaSH and zoonotic disease prevention. This counterintuitive finding has also been observed in other rural African contexts, suggesting that higher education does not guarantee context-specific health literacy [34,35]. This apparent paradox likely reflects a mismatch between general formal education (often urban-oriented and theoretical) and context-specific rural health needs, such as livestock-associated zoonotic risks, seasonal water scarcity, and pastoral sanitation challenges. Studies show that even educated individuals may lack targeted exposure to rural challenges, like livestock-associated health risks, inadequate water access, and sanitation issues, leading to overconfidence in generic knowledge while overlooking localized WaSH hazards, especially if they had not performed any targeted training [22,28,35,36]. This finding highlights the need for educational systems to integrate One Health and region-specific WaSH training in educational systems.

Age played a significant role, with individuals aged ≥30 years showing higher odds of good knowledge, suggesting that life experience and the traditional role of elders as knowledge transmitters contribute substantially to community-level awareness. This aligns with rural development literature highlighting the role of elders in sustaining community-based knowledge systems in African contexts, where they play a key role in transferring essential knowledge to younger generations [37].

The results show that knowledge of infectious diseases and their modes of transmission presents significant gaps, despite the high participation of the population in community meetings (85%) and prevention campaigns (over 70%). A substantial proportion of respondents do not recognise contaminated water as a cause of diarrhoea (24.6%) or underestimate the role of deceased persons and certain animal species (dogs, ruminants, poultry) in the transmission of zoonotic diseases. These misconceptions are consistent with findings from other rural sub-Saharan African contexts, where basic knowledge of zoonotic transmission remains limited even among populations in close contact with animals [25,33].

Overall, the results reinforce that effective communication strategies must be inclusive, context-specific, and gender-sensitive, combining interpersonal methods (e.g., home visits) with mass media (e.g., radio) and community platforms. Within a One Health framework, these strategies should integrate messages on human, animal, and environmental health, ensuring that educational programs address the unique epidemiological realities of rural settings. Such an integrated approach can enhance community engagement, improve the adoption of preventive behaviours, and ultimately strengthen resilience to health threats across all sectors.

### 4.5. Livestock

Livestock ownership reflects broader socioeconomic trends, with only 39% of families reporting ownership of any animals [38]. Statistical models revealed a strong association between owning small livestock, such as sheep, and good hygiene knowledge. This is likely due to more frequent interactions with veterinarians and animal health authorities during vaccination campaigns [5], particularly recent ones targeting small ruminants, which may have played a significant role in improving both animal health and hygiene awareness, as highlighted in Ghana, especially for the women [39].

Conversely, cattle ownership, often linked to traditional pastoral culture and, probably, to social status within the community, was associated with lower adoption of hygiene and sanitation measures. In many smallholder and pastoralist settings across sub-Saharan Africa, livestock, and cattle in particular, confer powerful social meanings and identities. While cattle represent wealth, social standing, and cultural heritage, this status dimension may also create barriers to changing entrenched practices, including those related to hygiene and sanitation [40].

Our findings confirm the high prevalence of livestock ownership in rural areas compared to urban settings, and suggest that ownership is often concentrated among a relatively small proportion of households. This pattern is consistent with previous observations in similar settings, where 15% of goat owners possessed 43% of the total goats and 15% of cattle owners possessed 60% of the total cattle [38,41,42,43]. Such disparities have important implications for targeting livestock-related interventions and ensuring equitable access to animal health resources. Pastoralism remains a critical component of resilience and sustainability in the region, providing economic stability and reinforcing cultural traditions. Programs that support small-scale pastoralism while promoting a more equitable distribution of livestock are essential to address disparities driven by Tropical Livestock Concentration, in which wealthier households retain ownership of the majority of animals [5,38]. Such imbalances can exacerbate social inequities and limit the capacity of poorer households to cope with crises.

KAP survey results also indicated that livestock owners who had received information from local authorities were more knowledgeable and more likely to implement preventive measures, highlighting the importance of targeted communication as an effective tool for improving disease control in the field. This underscores the importance of targeted veterinary communication in promoting effective biosecurity practices [5,28]. Vaccination emerged as the most widely implemented measure, likely reflecting the high visibility and logistical support of recent mass campaigns for small ruminants in the region. In contrast, the lower adoption of isolation of sick animals and cleaning of resting places points to both structural barriers, such as limited access to fencing, water, and cleaning supplies, and cultural perceptions of disease risk. In a previous study, Ugandan cattle farmers themselves reported financial and cultural constraints and highlighted the need for training and improved access to veterinary services [44]. Addressing these gaps through integrated animal–human health interventions could strengthen disease prevention and reduce zoonotic transmission, in line with the One Health approach.

### 4.6. Multivariate Analysis

The variables influencing knowledge, attitudes, and practices among the studied population differ significantly depending on whether they pertain to “sufficient” or “good” levels. Models predicting sufficient levels of knowledge, attitudes, and practices are characterised by a smaller number of key factors, which are relatively straightforward to identify and address. In contrast, achieving good levels involves greater complexity, requiring the integration of numerous socio-demographic, behavioural, and informational factors. This finding suggests that while sufficient levels can be reached by focusing on a few essential aspects, advancing to good levels necessitates a more sophisticated and integrated approach. Consequently, an effective approach to communication should combine various educational and communicative strategies, addressing a broad range of interconnected factors to achieve substantial and meaningful improvements.

### 4.7. Limits of the Study

This study encountered several limitations that may have influenced the results. Male interpreters were used during data collection, which could have affected the responses of female participants and potentially introduced bias; safety concerns restricted access to certain areas, possibly excluding more vulnerable populations and limiting the representativeness of the data; respondents may have over-reported hardships, perhaps in an attempt to attract future benefits, necessitating careful verification of the data; logistical issues, including impassable roads during the rainy season, hindered access to some communities, further complicating data collection efforts; finally, due to the history of livestock raiding in the region, some respondents may have deliberately underreported their livestock ownership to protect their assets, potentially leading to an underestimation of actual livestock distribution.

## 5. Conclusions

The present study focused on describing various aspects of the development in the Karamoja sub-region, Uganda. The objective was to understand the living conditions of the Karimojong population, the progress achieved over the years through development projects, the most effective means of receiving information, and the areas that require more attention. By conducting interviews with women and men, girls and boys, from different villages and settlements across the Moroto and Napak districts, the study highlighted how the efforts made by the government, local administration, and international organizations on issues such as water availability, access to sanitation facilities, information dissemination, and education on hygiene and infectious disease prevention, are gradually reaching acceptable levels, aligning with the national average.

However, the study also revealed areas for improvement in other aspects. Of particular concern is the declining trend in livestock possession and management, notably the best way to cope with both the frequent crises of the region and with climate change, which is having an enormous impact on the area. Another topic that requires attention is the implementation of received information on hygiene and prevention. Many people, despite receiving communications, do not translate this knowledge into actual daily life practices. On the other hand, a percentage of livestock owners have not received any information at all, and this group shows scarce knowledge on how to properly cope with hygiene and disease prevention measures.

Another important issue concerns waste management. Identifying sustainable solutions for waste disposal is urgent, as it is becoming an increasingly critical challenge with potential adverse effects on both public and environmental health, particularly in a One Health perspective.

Overall, the findings underscore the importance of lifestyle-related behaviours and increased public awareness as central determinants of health outcomes. Strengthening awareness-raising initiatives and promoting behavioural changes are therefore essential to ensure that improvements in the dissemination of WaSH infrastructure and information translate into sustained health benefits.

Lastly, to continue the development of the region and build on the remarkable progress achieved over the years, efforts should be made towards providing access to clean water for the entire population, fostering the empowerment of women and encouraging a shift towards greater gender equity within the community.

By addressing these interlinked factors, interventions can foster long-term improvements in health, social equity, and economic stability, contributing to a more sustainable and resilient future for the Karamoja sub-region. Further and complementary studies, adopting a One Health perspective, are warranted to integrate behavioural, environmental, and water-quality assessments and to better capture the evolution of WaSH practices and health outcomes over time.

## Figures and Tables

**Table 1:** Demographic characteristics of the survey participants in Moroto and Napak Districts, Karamoja Sub-Region, Uganda.

Demographic Variable	Women (n = 99) (%)	Men (n = 96) (%)	Total (n = 195)
Age	range: 16–80 years	range: 16–80 years	
Location	74 from villages (46.8) 25 from the city (67.6)	84 from villages (53.2) 12 from the city (32.4)	158 from villages 37 from Moroto city
Education Level			
No Schooling	25 (25.3)	20 (20.8)	45
Primary School Completed	35 (35.4)	32 (33.3)	67
Secondary School Completed	30 (30.3)	39 (40.6)	69
University Attended	9 (9.1)	5 (5.2)	14
Occupation			
Unemployed	22 (22.2)	12 (12.5)	34
Small-Scale Business	25 (25.3)	38 (39.6)	63
Agriculture	6 (6.1)	15 (15.6)	21
Selling Local Brew	10 (10.1)	1 (1.0)	11
Tailoring	8 (8.1)	6 (6.3)	14
Hairdressing	6 (6.1)	-	6
Selling Firewood/Charcoal	7 (7.1)	4 (4.2)	11
Local Administration Work	2 (2.0)	5 (5.2)	7
Stone Breaking	-	3 (3.1)	3

**Table 2:** Data regarding primary and secondary water sources, distance travelled for water collection, water treatment practices, and the role of women in water transportation in rural and urban areas (villages of Moroto and Napak districts and Moroto city). Statistical significance was reported where applicable.

Factor	Village (%)	City (%)	*p*-Value
Use of safe water sources	n = 158	n = 37	0.279
Handpumps/boreholes	115 (72.8)	25 (67.6)	
Public taps/standpipes	28 (17.7)	11 (29.7)	
Piped water	4 (2.5)	0 (0)	
Private boreholes	3 (1.9)	0 (0)	
Use of unsafe water sources	n = 158	n = 37	1.0
Rivers	8 (5.1)	1 (2.7)	
Access to secondary water sources	n = 158	n = 37	0.0217
Second borehole	59 (37.3)	8 (21.6)	
Public tap	12 (7.6)	7 (18.9)	
Surface water	11 (7.0)	1 (2.7)	
Private vendors	0 (0)	1 (2.7)	
No secondary source	76 (48.1)	20 (54.1)	
Distance travelled to the primary water source	n = 158	n = 37	0.0006
Less than 1 km	127 (80.4)	27 (45.9)	
1–1.5 km	21(13.3)	11 (29.7)	
>1.5–2 km	2 (1.3)	6 (16.2)	
>2–5 km	8 (5.1)	3 (8.1)	
Water transportation responsibility (more categories can collect water)	n = 154	n = 37	0.266
Women (mothers)	123 (79.9)	25 (67.6)	
Women (daughters)	68 (44.2)	8 (21.6)	
Men (fathers)	36 (23.4)	2 (5.4)	
Men (sons)	67 (43.5)	9 (24.3)	
Other	11 (7.1)	3 (8.1)	
Water treatment methods	n = 154	n = 37	0.496
Do not treat	91(57.6)	26 (70.3)	
Boil	54 (34.2)	10 (27.0)	
Boil and use of specific disinfectant products	5 (3.2)	0 (0)	
Use of specific disinfectant products	4 (2.5)	0 (0)	
Let stand and settle	4 (2.5)	1 (2.7)	

**Table 3:** Questionnaire responses regarding hygiene practices in the villages of Moroto and Napak districts and in Moroto city.

Hygiene Measure	Village (%)	City (%)	*p*-Value
Cleaning water containers regularly	n = 158	n = 37	0.136
Every time before filling	29 (18.4)	8 (21.6)	
Once a day	24 (15.2)	2 (5.4)	
At least once a week	92 (58.2)	19 (51.4)	
At least once a month	10 (6.3)	7 (18.9)	
Only when visibly dirty	1 (0.01)	0 (0)	
Never clean	3 (1.9)	1 (2.7)	
Use of soap for handwashing (more kind of materials can be used)	n = 158	n = 37	0.0006
Always use soap	137 (86.7)	33 (89.2)	
Ashes	83 (52.5%)	3 (8.1)	
Use only water	8 (5.1)	4 (10.8)	
Moments of handwashing	n = 158	n = 37	0.128
After defecation	129 (81.6)	32 (86.5)	
After urination	118 (74.5)	32 (86.5)	
After cooking	40 (25.3)	4 (10.8)	
After eating	111 (70.3)	26 (70.3)	
After giving hand	21 (13.3)	2 (5.4)	
After cleaning home	34 (21.5)	8 (21.6)	
Once back home	64 (40.5)	16 (43.2)	
Other	65 (41.1)	5 (13.5)	

**Table 4:** Data collected on sanitation practices in the villages of Moroto and Napak districts and in Moroto city.

Sanitation Measure	Village (%)	City (%)	*p*-Value
Excretal disposal	n = 158	n = 37	0.0003
Use of private latrines	64 (40.5)	13 (35.1)	
Use of public latrines	48 (30.4)	22 (59.5)	
Practice of open defecation	53 (33.5)	2 (5.4)	

**Table 5:** Data collected on waste management practices in the villages of Moroto and Napak districts and in Moroto city.

Waste Management	Village (%)	City (%)	*p*-Value
Waste disposal method	n = 158	n = 37	<0.0001
Burning near home	80 (50.6)	21 (56.8)	
Common pits	23 (14.6)	26 (70.3)	
Private pits	42 (26.6)	6 (16.2)	
Designated open areas	28 (17.7)	0 (0)	
Undesignated open areas	41 (25.9)	2 (5.4)	
Dumping into rivers	5 (3.2)	1 (2.7)	

**Table 6:** Communication and knowledge data collected by questionnaire in the villages in the Moroto and Napak districts and in Moroto city.

Communication/Knowledge Measure	Village (%)	City (%)	*p*-Value
Individuals with at least primary education	n = 158	n = 37	0.039
Male children	6 (10.9)	8 (14.5)	
Female children	1 (1.8)	0 (0)	
Fathers	26 (47.2)	4 (7.2)	
Mothers	21 (38.1)	16 (29.1)	
Grandfathers	0 (0)	0 (0)	
Grandmothers	1 (1.8)	0 (0)	
Ownership of electronic devices	n = 158	n = 37	0.126
Mobile phones	127 (80.4)	37 (100.0)	
Radios	79 (50.0)	29 (78.4)	
Televisions	18 (11.4)	11 (29.7)	
Preferred communication method	n = 158	n = 37	0.0009
Radio	90 (57.0)	20 (54.1)	
Community meetings	83 (52.5)	5 (13.5)	
Home visits	56 (35.4)	9 (24.3)	
Knowledge of diarrhoea causes (contaminated food)	135 (85.4)	12 (32.4)	
Knowledge of diarrhoea causes (contaminated water)	36 (22.8)	12 (32.4)	
Belief that bathing in a river cannot transmit diseases	20 (12.7)	4 (10.8)	
Belief that drinking directly from a well cannot transmit diseases	37 (23.4)	3 (8.1)	
Belief that insects cannot transmit diseases	5 (5.4)	2 (3.2)	
Belief that dogs cannot transmit diseases	22 (13.9)	7 (18.9)	
Belief that ruminants cannot transmit diseases	37 (23.4)	6 (16.2)	
Belief that chickens cannot transmit diseases	48 (30.4)	10 (27.0)	
Belief that drinking directly from a well is safe	121 (76.6)	34 (91.9)	
Belief that deceased persons cannot transmit diseases	51 (32.3)	6 (16.2)	
Participation in recent hygiene/disease prevention campaigns	142 (89.9)	27 (73.0)	

**Table 7:** Preventive measures considered useful by respondents for specific feared diseases.

Disease	Measure	%	Disease	Measure	%
Cholera			Ebola		
	Handwashing	56		Handwashing	67
	Proper cooking	27		Physical distance	64
	Keeping personal hygiene	23		Keeping personal hygiene	12
	Treatment of water	17			
	Proper conservation of food	11			
COVID-19			Tuberculosis		
	Handwashing	62		Physical distance	44
	Physical distance	62		Covering mouth and nose	38
	Covering mouth and nose	58		Handwashing	36
	Keeping personal hygiene	20		Keeping personal hygiene	26
	Vaccination	6			
	Avoid handshaking	3			
HIV/sexual transmissible diseases			Malaria		
	Usage of condoms	20		Sleeping under a net	30
	Be faithful to partner	16		Clean environment	22

**Table 8:** Summary of livestock ownership and water sources by animal type across the villages of Moroto and Napak districts and Moroto city.

Variable	Village (%)	City (%)	*p*-Value
Percentage of Respondents Owning Animals	68 (43% of respondents)	9 (24.3% of respondents)	0.04
Cattle Ownership (%)	30 (44.1% of the respondents owning animals)	5 (55.6% of the respondents owning animals)	0.43
Average number of Cattle per Owner	9	2.2	
Goat Ownership	37 (54.4)	6 (66.7)	0.34
Average number of Goats per Owner	9.9	3.3	
Sheep Ownership	7 (10.3)	2 (22.2)	0.79
Average number of Sheep per Owner	9.1	4.5	
Chicken Ownership	45 (66.2)	2 (22.2)	0.002
Average number of Chickens per Owner	10.2	5.5	0.32
Duck Ownership	6 (8.8)	0 (0)	0.23
Turkey Ownership	4 (5.9)	0 (0)	0.39
Rabbit Ownership	2 (2.9)	0 (0)	0.49
Water Source for Cattle			
-Surface Water	10 (33.3)	4 (80.0)	0.05
-Protected Springs	11 (36.7)	0 (0)	0.1
-Boreholes	9 (30.0)	0 (0)	>0.05
-Public Taps	0 (0)	1 (20)	0.13
Water Source for Small Ruminants			
-Boreholes	16 (23.1)	0 (0)	0.39
-Surface Water	14 (25.4)	3 (33.3)	0.69
-Public Taps	2 (3.6)	3 (33.3)	0.02
-Protected Springs	10 (18.2)	0 (0)	0.32
Water Source for Poultry			
-Boreholes	25 (45.4)	1 (11.1)	1
-Surface Water	8 (25.4)	1 (33.3)	0.35
-Public Taps	7 (12.7)	0 (0)	1
-Protected Springs (%)	5 (9.0)	0 (0)	1
Distance from Water Source			
-Boreholes/Public Taps within < 250 m (%)	31 (45.6)	3 (33.3)	0.19
-Protected Springs > 1 km (%)	3 (5.4)	0 (0)	0.54
-Surface Water < 1 km (%)	3 (5.4)	1 (11.1)	0.36
-Surface Water 1–2.5 km (%)	21 (38.2)	2 (22.2)	1
-Surface Water > 2.5 km (%)	12 (21.8)	0 (0)	1
Communications received from Authorities on Livestock Disease Prevention (%)	57 (83.8)	2 (22.2)	<0.001

**Table 9:** Multivariable logistic regression results for predictors of sufficient and good knowledge levels.

Outcome:			
Sufficient Knowledge			
Predictor	OR	95% CI	*p*-Value
Home visits	1.96	1.06–3.62	0.031
Employment	2.22	1.15–4.30	0.017
Good Knowledge			
Internet access	25.04	3.17–197.68	0.002
Home internet use	69.80	4.28–1138.04	0.003
Sheep ownership	16.89	1.54–185.64	0.021
Gender (women)	0.04	0.006–0.30	0.002
Age (≥30 years)	20.39	2.74–151.83	0.003
Higher education	0.01	0.001–0.14	<0.001

**Table 10:** Multivariable logistic regression results for predictors of sufficient and good Attitudes.

Outcome:			
Sufficient Attitudes			
Predictor	OR	95% CI	*p*-Value
Distance to water sources (>1 km)	3.56	1.57–8.11	0.002
Awareness of hygiene	0.32	0.13–0.83	0.020
Good Attitudes			
Higher education	6.89	3.12–15.20	<0.001
Owning livestock	6.96	2.90–16.70	<0.001
Community meetings	2.34	1.12–4.90	0.024
Urban residence (Moroto)	0.10	0.03–0.35	<0.001
Cattle ownership	0.14	0.05–0.43	<0.001

**Table 11:** Multivariable logistic regression results for predictors of sufficient and good Practices.

Outcome: Sufficient Practices			
Predictor	OR	95% CI	*p*-Value
Gender (female as reference)	0.36	0.16–0.83	0.017
Distance to water sources (>1 km)	6.10	2.72–13.68	<0.001
Radio ownership	3.529	1.45–8.59	0.005
Good Practices			
Gender (women)	4.49	1.43–14.08	0.010
Higher education	4.17	1.36–12.75	0.012
Poultry ownership	4.61	1.49–14.22	0.008
Home visits	30.78	8.07–117.42	<0.001
Owning a radio	15.72	4.04–61.20	<0.001
Internet access	21.30	6.07–74.70	<0.001
Distance to water sources (>1 km)	0.20	0.06–0.70	0.011

## Data Availability

The data presented in this study are available upon request from the corresponding author. The data is not publicly available due to privacy and ethical restrictions.

## Supplementary Materials

The following supporting information can be downloaded at https://www.mdpi.com/article/10.3390/epidemiologia7020052/s1, Questionnaire S1.
