# Supplementary material for: Epidemiological Survey on Water, Sanitation, and Hygiene (WaSH) in Uganda’s Karamoja Sub-Region, Using a KAP Questionnaire Within a One Health Framework
Source: Epidemiologia (Basel). 2026 Apr 7;7(2):52. doi: 10.3390/epidemiologia7020052 (PMC13115495; doi:10.3390/epidemiologia7020052)
Supplement: Supplementary file 1 [file epidemiologia-07-00052-s001.zip › epidemiologia-4037240-supplementary.pdf]

WaSH in Karamoja to prevent infectious diseases transmission

Date

yyyy-mm-dd

hh:mm

Place of the interview

- C&D center
- Moroto city
- Village
- Other

If "other", where?

Respondent's village name

District

- Moroto
- Other

If "other", where?

Site name

Language of the interview

- English
- Karimojong

Respondent's name

Respondent's gender

- Male
- Female

Respondent's age

Respondent's role in the household (family)

- Grandfather
- Grandmother
- Father
- Mother
- Female child/daughter
- Male child/son
- Other

Do you have a job?

- Yes
- No

Respondent's job

If "Other", what?

Respondent's activity or role in the village

Respondent's education level

- No instruction
- Primary school
- Secondary school
- University

Respondent's ethnic affiliation

- Karimojong
- Iteso
- Acholi
- Kenyan
- Alur
- Baganda
- Other

If "Other", what?

Number of sons

Age of sons

Number of daughters

Age of daughters

Does your family own livestock?

- Yes
- No

What livestock do you have?

- [ ] Cattle
- [ ] Swine
- [ ] Goat
- [ ] Sheep
- [ ] Donkey
- [ ] Chicken
- [ ] Turkey
- [ ] Rabbit
- [ ] Ducks

Number of cattle

____

Number of swine

____

Number of goat

____

Number of sheep

____

Number of donkey

____

Number of chicken

____

Number of turkey

____

Number of rabbit

____

Number of ducks

____

Who is responsible for the cattle management?

- [ ] Grandfather
- [ ] Grandmother
- [ ] Father
- [ ] Mother
- [ ] Male child/son
- [ ] Female child/daughter
- [ ] Uncle
- [ ] Aunt
- [ ] Friend
- [ ] Cousin

Who is responsible for the livestock (except cattle) management?

- [ ] Grandfather
- [ ] Grandmother
- [ ] Father
- [ ] Mother
- [ ] Male child/son
- [ ] Female child/daughter
- [ ] Uncle
- [ ] Aunt
- [ ] Friend
- [ ] Cousin

Where do you get your drinking water?

- ☐ Handpumps/boreholes
- ☐ Public tap/standpipe
- ☐ Pipes connected to the house
- ☐ Water seller/kiosks
- ☐ Rainwater collection
- ☐ Protected spring
- ☐ Surface water
- ☐ Other

If "other", where?

____

Who else has access to the primary water source?

- ☐ Villagers
- ☐ Anyone
- ☐ No one other than the household
- ☐ Other

If "other", who?

____

How much do you pay for your water per month on average?

____

Do you sometime take water from a secondary water source?

- ○ Handpumps/boreholes
- ○ Public tap/standpipe
- ○ Pipes connected to the house
- ○ Water seller/kiosks
- ○ Rainwater collection
- ○ Protected spring
- ○ Surface water
- ○ Other
- ○ No

If "other", where?

____

How do you treat the water before consumption?

- [ ] Do not treat it
- [ ] Let it stand and settle
- [ ] Boil it
- [ ] Expose it to sunlight
- [ ] Use disinfectant products
- [ ] Filter it
- [ ] Other

If "other", how?

____

Why you don't treat it?

____

What source of water do you use for livestock?

- [ ] Handpumps/boreholes
- [ ] Public tap/standpipe
- [ ] Protected spring
- [ ] Surface water
- [ ] Pipes connected to the house
- [ ] Other

If "other", what?

____

What is the distance to the primary water source for human consumption from household in meters?

____

What is the distance to the secondary water source for human consumption from household in meters?

____

What is the distance to water source for livestock consumption from animals site in meters?

____

How many times a day does the household transport water from the water source?

- ◯ Not every day
- ◯ Once a day
- ◯ Twice a day
- ◯ Three times a day
- ◯ Four times a day
- ◯ Five times a day
- ◯ More than five times a day

In what kind of container do you keep the water at home?

- ☐ Open jar
- ☐ Close jar
- ☐ Open jerry can
- ☐ Close jerry can
- ☐ Open bucket
- ☐ Close bucket
- ☐ Open container
- ☐ Close container
- ☐ Other

If "other", where?

____

In what container is the water being transported from the source to the household?

- ☐ Bucket
- ☐ Jerry can
- ☐ Through pipe system
- ☐ Other

If "other", what?

____

Who usually collects water from your household?

- [ ] Father
- [ ] Mother
- [ ] Male child/son
- [ ] Female child/daughter
- [ ] Other

If "other", who?

Is the current water supply/source accessible and reliable throughout the year?

- ( ) Yes
- ( ) No

If no, why?

- [ ] Sometimes it breaks
- [ ] Water shortages during dry season
- [ ] Water shortages during rainy season
- [ ] Water source too far
- [ ] Major source are controlled by land owner and limited access
- [ ] Conflict related to the use of water source
- [ ] Too dangerous to get the water
- [ ] Waiting time too long
- [ ] Don't have enough storage
- [ ] Limitation of volume of water that can be collected at water point
- [ ] Other

If "other", what?

How often do you clean the drinking water containers?

- ( ) Never
- ( ) Every time they are used
- ( ) Every day
- ( ) At least once a week
- ( ) At least once a month
- ( ) Other

If "other, what?

How are the drinking water containers cleaned?

- ☐ Specific commercial product
- ☐ Tissue/sponge
- ☐ Rocks or sand and shaking
- ☐ Soap
- ☐ Other

If "other", how?

What kind of facility does your household use for excreta disposal?

- ☐ Communal latrine
- ☐ Household latrine
- ☐ Open defecation
- ☐ Plastic bag
- ☐ Plastic pot
- ☐ Other

If "other", what?

Where do you dispose of your solid waste?

- ☐ Burn it
- ☐ Bury it
- ☐ Communal pit
- ☐ Designated open area
- ☐ Household pit
- ☐ Undesignated open area
- ☐ Other

If "other", how?

What do you use as cleaning material for hand washing?

- [ ] Water only
- [ ] Soap
- [ ] Ash
- [ ] Sand
- [ ] Other

If "other", what?

Why no soap?

- [ ] Run out of soap
- [ ] Can't afford it
- [ ] Soap unavailable in the area
- [ ] Soap is unnecessary
- [ ] Don't like soap
- [ ] Other

If "other", why?

Is there a specific hand washing device/station in the household?

- ○ Basin or bucket
- ○ Pouring device
- ○ No

When do you clean your hands?

- ☐ Before eating
- ☐ Before cooking
- ☐ After defecation
- ☐ After urination
- ☐ After touching livestock/domestic animals
- ☐ After touching animal's stool
- ☐ After giving the hand to someone else
- ☐ Before breastfeeding
- ☐ Before feeding children
- ☐ After handling a child's stool
- ☐ When coming back home
- ☐ After cleaning home
- ☐ Other

If "other", when?

____

How often do you shower/bath?

- ◯ More than once a day
- ◯ Once a day
- ◯ Multiple times a week
- ◯ Once a week
- ◯ Once a month
- ◯ Never
- ◯ Other

If "other", how much?

____

Do you collect enough water to meet your household needs?

- ◯ Yes
- ◯ No
- ◯ Not always

Would you collect more water daily if the source would be nearer?

- ◯ Yes
- ◯ No

Since when do you use your water source?

- ◯ Since last year
- ◯ Since the last 5 years
- ◯ Since the last 10 years
- ◯ Since more than 10 years

What was the previous water source?

- ◯ Handpumps/boreholes
- ◯ Public tap/standpipe
- ◯ Pipes connected to the house
- ◯ Water seller/kiosks
- ◯ Rainwater collection
- ◯ Protected spring
- ◯ Surface water

How distant was the previous water source (in meters)?

____

Why you changed your water source?

- ☐ Nearer water source has been constructed or rehabilitated
- ☐ Safer water source has been constructed or rehabilitated
- ☐ Previous water source is no more available
- ☐ Other

If "Other", why?

____

Do you feel like you have more free time than before since you use the actual water source?

- ◯ Yes
- ◯ No

Did you start a new activity since you use your actual water source?

- ◯ Yes
- ◯ No

If "yes", what?

Did your household's social condition improve since the construction of the actual water source?

- ○ Yes
- ○ No

If you had more free time, how would you spend it?

- ○ Getting further education
- ○ Start a new economic activity
- ○ Meet with family or friends more often
- ○ Relax
- ○ Other

If "other", what?

Would you consider getting more livestock if more reliable water sources are available?

- ○ Yes
- ○ No

If "No", why?

What are in your opinion the best means to receive hygiene and health communication?

- ☐ Home visit
- ☐ Community meeting
- ☐ Radio
- ☐ Internet
- ☐ Printed flyers
- ☐ Speaker vehicle
- ☐ From church
- ☐ Other

If "other", what?

Who in the household is able to read?

- [ ] Grandfather
- [ ] Grandmother
- [ ] Father
- [ ] Mother
- [ ] Female child/daughter
- [ ] Male child/son
- [ ] Other

If "other", who?

Do you have a functioning electronic device in your household?

- [ ] Radio
- [ ] Mobile phone
- [ ] Tv
- [ ] None

Do you have access to the internet?

- ○ Yes
- ○ No

Do you think that having access to clean water is a sign that God sees and loves you?

- ○ Yes
- ○ No

Having access to clean water provides you hope for the future?

- ○ Yes
- ○ No

Did you receive or attend awareness sessions related on good hygiene practices?

- ○ Yes
- ○ No

What topics are covered?

- [ ] Importance of personal hygiene
- [ ] Importance of safe excreta disposal and usage of latrine
- [ ] Importance of handwashing using soap during key times
- [ ] Diseases caused by poor WaSH practices
- [ ] Household water treatment
- [ ] Importance of cleaning home
- [ ] Other

If "other", what?

___

What are the most dangerous infectious diseases among people in your opinion?

- [ ] Malaria
- [ ] Rabies
- [ ] Cholera
- [ ] Ebola
- [ ] Covid-19
- [ ] Tuberculosis
- [ ] HIV
- [ ] Influenza
- [ ] Other

If "other", what?

___

Did you receive any communication about measures to prevent the spread of infectious diseases in people?

- ○ Yes
- ○ No

Could you tell me which measures can prevent the spread of infectious diseases among people?

- [ ] Handwashing during key times
- [ ] Proper cooking of food
- [ ] Proper conservation of food
- [ ] Treatment of water before consumption
- [ ] Maintaining physical distance with someone sick
- [ ] Covering mouth and nose
- [ ] Other

If "other", what?

What are the ways that people can get diarrhea?

- [ ] Through contaminated or undercooked food
- [ ] Through contaminated water
- [ ] From flies
- [ ] From contact with someone sick with diarrhea
- [ ] From contact with animals sick with diarrhea
- [ ] From swimming/bathing in surface water
- [ ] Don't know
- [ ] Other

If "other", what?

What among these do you think can transmit infectious diseases to humans?

- [ ] Insects
- [ ] Bathing in rivers
- [ ] Drinking water directly from the boreholes
- [ ] Cattle and goat
- [ ] Dogs
- [ ] Poultry
- [ ] Dead people

Did you receive any communication about measures to prevent the spread of infectious diseases among animals?

- ( ) Yes
- ( ) No

Could you tell me which measures can prevent the spread of infectious diseases among animals?

- [ ] Vaccination
- [ ] Providing safe water
- [ ] Isolating sick animals
- [ ] Avoiding contact between livestock and wild animals
- [ ] Avoiding mixing animals from different species
- [ ] Avoiding overpopulation of animals
- [ ] Hygiene in their resting place
- [ ] Providing proper feed
- [ ] Other

If "other", what?

Do you know a fairytale or traditional legend related to water?
